# Nursing theories in the care of stroke patients: a scoping review

**DOI:** 10.1590/0034-7167-2022-0791

**Published:** 2023-10-06

**Authors:** Josefa Nayara de Lima, Layane Ribeiro Lima, Edilma Gomes Rocha Cavalcante, Glauberto da Silva Quirino, Woneska Rodrigues Pinheiro

**Affiliations:** I Universidade Regional do Cariri. Crato, Ceará, Brazil

**Keywords:** Nursing Theory, Stroke, Nursing Care, Nursing, Review, Teoría de Enfermería, Accidente Cerebrovascular, Atención de Enfermería, Enfermería, Revisión, Teoria de Enfermagem, Acidente Vascular Cerebral, Cuidados de Enfermagem, Enfermagem, Revisão

## Abstract

**Objectives:**

to map and synthesize nursing theories and conceptual frameworks that have been applied in the practice of nursing care for stroke patients in hospital settings.

**Methods:**

a scoping review was conducted in October 2022 using the MEDLINE (accessed via PubMed), CINAHL, Scielo, and Web of Science databases, following The Joanna Briggs Institute guidelines.

**Results:**

nine studies incorporated six nursing theories and three conceptual frameworks, which were employed to enhance stroke patient care. The objective of these theories and conceptual frameworks was to facilitate the identification of the patient’s psychobiological, psychosocial, and psychospiritual needs, elucidate the nurse’s role and expand their perspective on rehabilitation, and acknowledge the survivor’s process of transition.

**Final Considerations:**

this mapping exercise identified major nursing theories, middle-range theories, and conceptual frameworks applied to the care of stroke patients.

## RESUMO

**Objetivos:**

mapear e sintetizar teorias de enfermagem e estruturas conceituais que foram aplicadas na prática do cuidado de enfermagem ao paciente vítima de Acidente Vascular Cerebral na atenção hospitalar.

**Métodos:**

revisão de escopo realizada em outubro de 2022 nas bases de dados MEDLINE (acesso via PubMed), CINAHL, Scielo e *Web of Science*, conforme o The Joanna Briggs Institute.

**Resultados:**

os nove estudos apresentavam seis teorias de enfermagem e três estruturas conceituais que foram utilizadas para apoiar a assistência ao paciente com Acidente Vascular Cerebral. Essas teorias e estruturas conceituais tinham como objetivo facilitar a identificação das necessidades psicobiológicas, psicossociais e psicoespirituais do paciente, esclarecer o papel e ampliar a visão do enfermeiro sobre a reabilitação, e reconhecer o processo de transição do sobrevivente.

**Considerações Finais:**

este mapeamento permitiu identificar grandes teorias, teorias de médio alcance e estruturas conceituais de enfermagem aplicadas ao cuidado ao paciente com Acidente Vascular Cerebral.

## RESUMEN

**Objetivos:**

mapear y sintetizar teorías de enfermería y estructuras conceptuales que se aplicaron en la práctica del cuidado de enfermería al paciente víctima de Accidente Cerebrovascular en atención hospitalaria.

**Métodos:**

revisión de alcance realizada en octubre de 2022 en las bases de datos MEDLINE (acceso a través de PubMed), CINAHL, Scielo y *Web of Science*, siguiendo los lineamientos del Instituto Joanna Briggs.

**Resultados:**

los nueve estudios presentaron seis teorías de enfermería y tres estructuras conceptuales que se utilizaron para respaldar la atención al paciente con Accidente Cerebrovascular. Estas teorías y estructuras conceptuales tenían como objetivo facilitar la identificación de las necesidades psicobiológicas, psicosociales y psicoespirituales del paciente, clarificar el papel y ampliar la visión del enfermero sobre la rehabilitación, y reconocer el proceso de transición del sobreviviente.

**Consideraciones Finales:**

este mapeo permitió identificar grandes teorías, teorías de alcance medio y estructuras conceptuales de enfermería aplicadas al cuidado del paciente con Accidente Cerebrovascular.

## INTRODUCTION

Cerebrovascular diseases, particularly stroke, are a significant cause of adverse neurological outcomes, deaths, and disabilities^(1)^. Globally, stroke was the second leading cause of death in 2019, with 12.2 million incident cases, 101 million prevalent cases, and 55 million deaths^(1)^. In Brazil, stroke was responsible for 99,010 deaths in 2020, remaining the country’s second leading cause of death^(2)^.

Stroke is characterized by a focal neurological impairment of vascular origin, which can be global with a sudden onset and a duration of more than 24 hours, often leading to death. It is the second leading cause of disability in adults worldwide^(3)^. Generally, there are two main types of strokes: ischemic and hemorrhagic, both resulting in hypoxia and brain tissue damage^(4)^.

It is estimated that 74% of people survive stroke, and 57% of them will require care provided by family members. However, even with available healthcare technologies, recovery after a stroke is complex and involves social, biomedical, and psychological aspects related to health, well-being, and quality of life, with the support of the nursing team^(5)^.

The nursing team plays a fundamental role in caring for stroke patients. When there is a deep understanding of the patient’s clinical condition and the alterations caused by the accident, the care can be directed to meet the specific needs of the patient^(6)^.

Currently, nursing is being recognized as a profession with its own knowledge, and nurses need to base their practices on scientific knowledge supported by various governmental organizations and the profession worldwide. These organizations emphasize the importance of professionals capable of adopting preventive, curative, rehabilitative, and health-promoting measures in the population. Theories play a fundamental role in this science, providing theoretical and practical support for nurses^(7)^.

Nursing theories are based on specific phenomena within the profession. They generate ideas that point to the essence of practice, offering opportunities for a broader and more critical understanding. They make a relevant contribution, particularly by expanding the understanding of the concept of good nursing practices^(8)^. They are useful in explaining, describing, and prescribing measures in the care practice, supporting nursing knowledge and practices. Therefore, the construction, validation, and discussion of nursing theories are essential to guide the advancement of nursing as a science and profession^(9)^.

In this context, nursing theories/theoretical models can contribute to the provision of competent care, improving quality, as they enable nurses to articulate what they do with patients and why they do it. There is evidence that nursing care developed from a theoretical perspective improves outcomes, demonstrating the usefulness of theoretical practice^(10)^.

However, studies specifically addressing and presenting nursing theories focused on the care of stroke victims are still scarce. Existing literature, which is not specific to nursing, and data related to the use of theories in practical nursing care contexts, especially regarding stroke, are limited. Additionally, incorporating theories into nursing practice is a recurring challenge^(9)^.

The findings of this study can provide valuable insights for knowledge production in the field and assist nurses in organizing their actions by embracing and appropriating theories, promoting standardized and systematic care. The quality of nursing care can contribute to increasing the survival rate of people affected by stroke and, above all, preventing sequelae.

Furthermore, this knowledge can contribute to the education of new professionals, supporting the development of patient-centered care, considering its crucial role in ensuring quality care. The data can also help prioritize and value the patient’s experience in the recovery process, preserving their autonomy.

## OBJECTIVES

To map and synthesize nursing theories and conceptual frameworks that have been applied in the practice of nursing care for stroke patients in hospital settings.

## METHODS

### Ethical considerations

Ethics committee approval was waived as this study is a review using publicly available data and does not involve human subjects.

### Study Design

This is a scoping review based on the methodology of the Joanna Briggs Institute (JBI) (11) and structured according to the Preferred Reporting Items for Systematic Reviews and Meta-Analyses extension for Scoping Reviews (PRISMA-ScR)^(12)^ guidelines. The study was conducted in the following stages: identification of the research question, identification of relevant studies, study selection, data analysis, synthesis, and data presentation^(12)^.

### Identification of the research question

The strategy used to guide the construction of the research question and the choice of descriptors was based on the PCC mnemonic: Population (P) - Stroke patients, Concept (C) - Nursing theories, and Context (C) - Nursing care in a hospital setting. This resulted in the question: What nursing theories and conceptual frameworks are used in the practice of nursing care for stroke patients in a hospital setting?

### Identification of relevant studies

The search was conducted by two researchers in October 2022 in three stages. Initially, a cross-reference of the controlled descriptors Medical Subject Headings (MeSH): nursing theory AND stroke was used in the databases Medical Literature Analysis and Retrieval System Online (MEDLINE^®^) (accessed via PubMed) and Cumulative Index to Nursing and Allied Health Literature (CINAHL). Titles, keywords, descriptors, and abstracts of the studies that were relevant to the review’s topic were explored. Subsequently, full-text reading was conducted.

In the second stage, using the selected descriptors, the following search strategies were constructed with MeSH terms: nursing theory AND stroke AND nursing care; nursing theory AND stroke nursing; Conceptual Framework AND stroke AND nursing care; Specific situation theory AND stroke. These strategies were applied to the following databases: MEDLINE^®^ (via PubMed), CINAHL (via EBSCO platform), Web of Science, and Scientific Electronic Library Online (Scielo).

The same strategies were used to search for gray literature through the Nursology website. To gather information from the Catalog of Theses and Dissertations of the Coordination for the Improvement of Higher Education Personnel (CAPES) and Google Scholar, a strategy was developed based on the Health Sciences Descriptors (DeCS): nursing theories AND stroke AND nursing care. Additionally, a search was conducted for new documents in the reference lists of the selected sources to be included in the study.

A total of 1,403 studies were identified in the searched data sources, and the sample consisted of nine studies. For inclusion in this review, literature published in any language, available online, in full-text format, without a temporal restriction, and focusing on nursing theories used for stroke patient care in a hospital context, including gray literature data, was selected. Duplicate studies, editorials, and conference proceedings were excluded.

### Study selection

The studies were independently selected by two researchers. After the search, the studies were exported to the Rayyan QCRI selection platform. Rayyan supports authors of systematic and scoping reviews in conducting their studies quickly and efficiently by allowing the export of articles from a specific database to the program and presenting titles and abstracts with the blinding of the assisting researcher, ensuring reliability in study selection, accuracy, and methodological rigor^(13)^.

**Figure 1:**
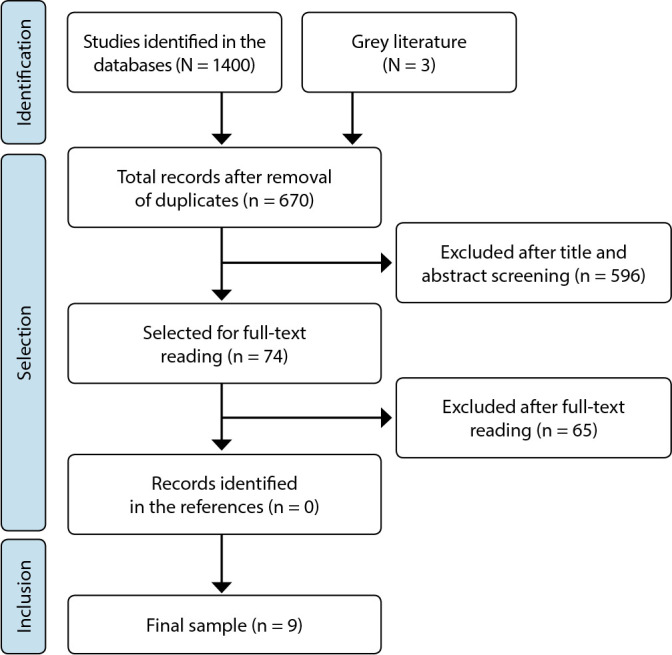
Study selection flowchart adapted from PRISMA-ScR and JBI recommendations, Crato, Ceará, Brazil, 2022

Initially, duplicate studies were removed. Then, titles and abstracts were evaluated, followed by a full-text assessment, based on the eligibility criteria. Any discrepancies in the evaluations were resolved by a third author. The entire selection process is depicted in the flowchart (Figure 1), based on PRISMA-ScR, following the JBI recommendations^(14)^.

### Data analysis

The studies selected to compose the review sample were evaluated according to the levels of evidence established by the Agency for Healthcare Research and Quality (AHRQ) of the United States. In this case, evidence was classified into seven levels, taking into consideration the production of nursing knowledge from quantitative and qualitative research: level 1, systematic review or meta-analysis of randomized controlled trials/clinical guidelines that contain the aforementioned review studies; level 2, well-designed randomized controlled trials; level 3, controlled clinical trial but without randomization; level 4, well-designed case-control or cohort studies; level 5, systematic review of descriptive and qualitative studies; level 6, descriptive or qualitative studies; and level 7, expert opinion^(15)^.

The studies were analyzed by two independent evaluators, and the data were extracted using a form divided into two dimensions: the first aimed at characterizing the included studies (country/year of publication, title, type of study, objective) and the second dimension allowed the extraction of information on nursing theories identified (type of theory, application in the study, main concepts used, outcome and applications in nursing care). The form used to collect data was adapted from the instrument used in Dantas’s study^(7)^.

The extracted information was organized in an electronic spreadsheet using Microsoft Office Excel^®^ 365, which allowed for the description of the results. A descriptive synthesis of the findings was conducted, which were presented in charts to provide better visualization and understanding of the obtained data. The discussion aimed to reflect on the findings according to the available literature on the subject.

## RESULTS

The search resulted in 1,403 records, with 538 in MEDLINE, 227 in CINAHL, 623 in Web of Science, 12 in Scielo, two in the Catalog of Theses and Dissertations from CAPES, and one in Google Scholar. Among these, 733 were duplicates, 596 did not contain subject descriptors in the title and abstract, and 65 did not meet the eligibility criteria, distributed as follows: eight did not involve the target population, 19 did not address a nursing construct, 32 were outside the hospital context, and six did not have available text. Only nine studies remained, which compose the final sample, as no records were identified in the references of the included studies. Of this total, two studies were from grey literature.

Chart 1 presents the characterization of the studies according to the year of publication, country, study type, objective, and context in which they were developed. It was found that the studies occurred between 2001 and 2021, with four publications from Brazil, two from China, and one each from Australia, Norway, and the United States. Regarding the hospital context in which the studies were developed, it was observed that one considered the Intensive Care Unit, another the emergency unit, four mentioned hospital units specialized in stroke, and two only mentioned “hospital unit”.

**Chart 1:** Characterization of studies composing the sample, Crato, Ceará, Brazil, 2022

Characterization of included studies					
**ID** **Code**	**Country / Year**	**Title**	**Study Type /** **LE**	**Objective**	**Context**
S1^(16)^	Brazil 2011	Systematized nursing care for individuals with stroke according to the theory of basic human needs	Qualitative study Level 6	Presenting the operationalization of Nursing Care Systematization (NCS) for a patient with stroke	Intensive Care Unit
S2^(17)^	China 2021	Application Value of Rehabilitation Nursing in Patients with Stroke Based on the Theory of Interactive Standard: A Randomized Controlled Study.	Non-randomized clinical trial Level 3	Exploring the value of applying rehabilitation nursing based on the theory of interactive patterns in patients with stroke	Hospital unit for stroke recovery
S3^(18)^	Australia 2013	Development and preliminary testing of a framework to evaluate patients’ experiences of the fundamentals of care: a secondary analysis of three stroke survivor narratives	Qualitative study Level 6	Develop and test a framework that describes the interrelation of three key dimensions (physical, psychosocial, and relational) in the provision of care to patients.	Intensive care
S4^(19)^	China 2021	The combinative effects of Orem self-care theory and PDCA nursing on cognitive function, neurological function and daily living ability in acute stroke	Non-randomized clinical trial Level 3	Explore the combined effects of Orem’s Self-Care Theory and PDCA nursing on cognitive function, neurological function, and daily living ability in patients.	Hospital unit
S5^(20)^	Brazil 2016	Application of Callista Roy’s nursing theory to patients with stroke	Descriptive study Level 6	Report the experience of applying the nursing process implemented in light of Callista Roy’s Theory to a patient affected by stroke.	Emergency unit
S6^(21)^	Norway 2010	The Role of Nursing in the Rehabilitation of Stroke Survivors An Extended Theoretical Account	Ethnographic study Level 6	Propose a revised and expanded theoretical account of the nursing role in stroke rehabilitation.	Specialized Stroke Unit
S7^(22)^	United States 2001	A Framework for Care During the Stroke Experience	Literature review Level 7	Guide the selection and design of nursing activities that will facilitate the health of individuals with stroke and their families.	Hospital Unit
S8^(23)^	Brazil 2017	Care pathway for individuals with stroke: from onset to rehabilitation	Qualitative study Level 6	Describe the trajectory followed by individuals with Stroke and identify the significant findings along this pathway.	Specialized Stroke Care Unit
S9^(24)^	Brazil 2017	Nursing guidelines for hospital discharge of patients with stroke based on Wanda Horta	Descriptive study Level 6	Develop nursing guidelines for the hospital discharge of patients affected by Stroke based on basic human needs.	Specialized Stroke Care Unit

ID - Identification; S - Study; LE - Level of Evidence; PDCA - Plan, Do, Check, Act.

Regarding the levels of evidence, the studies presented low levels, mostly being qualitative and descriptive. Only two studies presented a higher level of evidence, demonstrating the need for the development of research with greater potential for scientific evidence that can guide decisions in this context.

In Chart 2, the results related to the theories are characterized, considering the title, the type of theory according to its scope, the description of how the study used the theory, the main concepts used, and the approach of the applicability for nursing care to the patient with stroke. It was identified that three studies used a middle-range theory, with two of them using the theory of basic human needs, while in three other studies, a grand theory was applied. It is noteworthy that three studies addressed the development of conceptual frameworks as a focus on improving nursing care for hospitalized patients due to stroke.

**Chart 2:** Description of identified nursing theories or conceptual frameworks, Crato, Ceará, Brazil, 2022

Description of nursing theories				
**ID Code**	Title/Theory Type^*^	**How the theory was used**	**Main applied concepts**	**Outcome and applicability for nursing care to the patient with stroke**
S1	Human Basic Needs Theory (HBNT)/ Middle-range theory	Used the theory to underpin the Nursing Care Systematization (NCS)	Patient needs; environment; nursing	Identification of psychobiological, psychosocial, and psycho-spiritual needs and planning appropriate interventions.
S2	Interactive Systems Theory/ Grand theory	The nursing team received training based on the theory for the implementation of interactive nursing rehabilitation care	Patient’s personal, interpersonal, and social systems; Nursing process	atients who received interactive nursing care achieved better neurological function and improved quality of life; Provides nurses with a clear understanding of recovery goals.
S3	Conceptual framework/Not applicable	The framework allowed for the identification of factors that could explain the care experiences of stroke survivors, including acute hospital care	Fundamentals of care; Physical, psychosocial, and relational dimensions	Useful as a predictive framework to indicate when care will not be integrated or person-centered Demonstrates the interaction of physical, psychosocial, and relational experiences for care to be positively experienced.
S4	Theory of self-care/ Grand theory	It underpinned the implementation of the PDCA (Plan-Do-Check-Act) strategy to enhance care for patients with acute stroke.	Self-care; Patient; Care deficit	It can actively improve patients’ cognitive function, neurological status, and daily living capacity. Empowerment of self-care; Assists the nursing team in formulating and implementing plans to enhance the care process.
S5	Roy’s Adaptation Model/ Grand theory	The theory was used to support the application of the nursing process.	Nursing diagnosis; Goal setting; Intervention; Evaluation.	It contributes to effective nursing care for patients affected by stroke and emphasizes the importance of stimuli that trigger responses requiring patient adaptation.
S6	Conceptual framework/ Not applicable	The framework allowed for the identification of the nursing role in stroke rehabilitation based on a review of recent research on stroke.	Fundamentals of nursing care for patients in stroke rehabilitation.	It elucidates the nurse’s role in stroke rehabilitation and expands the focus of nursing care for patients with stroke to include the acute and early rehabilitation phase.
S7	Conceptual framework/ Not applicable	The framework allowed for the identification and organization of nursing care in the context of stroke.	Fundamentals of nursing care.	It is useful for increasing nurses’ knowledge about the stroke experience and helping them recognize and articulate their roles in the care of individuals with stroke.
S8	Theory of Transitions by Meleis/ Mid-Range	The theory was used to deepen the understanding of the patient’s transition after stroke.	Nature of the transition; Conditions of the transition; Patterns of response to the transition.	Recognition of the stroke transition process and the necessary care; integration of the patient with the hospital environment and routine.
S9	Theory of Basic Human Needs/ Mid-Range	The theory was used to develop nursing guidelines for hospital discharge.	Patient needs; Environment; Nursing	Identifies the psychobiological, psychosocial, and psycho-spiritual demands during hospitalization that have an impact on home care.

* According to scope; ID - Identification; S - Study.

It was possible to identify that the theories and conceptual frameworks were used to support nursing care aimed at the recovery of stroke patients, considering various aspects of the journey: facilitating the identification of psychobiological, psychosocial, and psycho-spiritual needs; clarifying the role and expanding the nurse’s vision of the rehabilitation process; recognizing the survivor’s transition and adaptation process.

## DISCUSSION

Nursing possesses distinct knowledge that should be incorporated into an increasingly complex world of interprofessional practices. Therefore, the need to utilize nursing theories is understood, as they provide a framework through which thoughts about nursing phenomena can be organized. These theories contribute to the description of care patterns, serve as the basis for implementing interventions, and generate new knowledge for use in practice^(25)^.

Stroke is a prominent event in the epidemiological landscape, reinforcing the need for timely nursing care, whether in the acute phase of the disease or during rehabilitation, focused on prevention and control of complications^(26)^. Such care is also directed towards managing the hospital environment and organizing the healthcare team^(27)^, as this set of actions addresses a substantial portion of patient demands.

Thus, the results indicate the use of grand theories and mid-range theories to underpin nursing care for patients affected by stroke. Mid-range theories are predominant in contemporary nursing practice, as they are often employed to describe mechanisms and processes that explain observed phenomena in daily practice or other health-related contexts, rather than describing natural relationships^(28)^. On the other hand, grand theories are challenging to apply to the empirical world and serve as worldviews and philosophies, contributing to the theoretical development of nursing^(8)^.

The findings suggest that both types of theories were applicable and relevant to the researched context, as grand theories facilitated care pathways, while mid-range theories addressed more specific demands of nursing care for stroke patients.

In addition to theories, conceptual frameworks were identified that made explicit the foundations of care directed towards specific situations in the post-stroke context. In this scenario, a conceptual framework serves as a valid construct, as it has the ability to identify gaps in understanding a phenomenon or problem^(29)^.

The most frequent theory was Wanda de Aguiar Horta’s Theory of Basic Human Needs, which contributed to managing the psychobiological, psychosocial, and psycho-spiritual needs of individuals affected by stroke. This finding is similar to a bibliometric study conducted in Brazil, where the theory was found to be the second most present in developed theses^(9)^.

The prioritized demands of individuals with stroke can be found within the conceptual framework of Horta’s theory, including the needs for intensive care in the acute phase of the disease. During this specific period, the focus will be on interventions aimed at maintaining biological functions, such as vital sign monitoring, airway assessment, and nutrition^(30)^. Furthermore, it will be necessary to incorporate care to manage multidimensional aspects, such as cognitive and emotional aspects, which are largely unmet^(31)^.

Such care needs can be visualized by the nurse from a theoretical perspective facilitated by the use of a theory, as evidenced by the results indicating that theoretical constructs enable a clearer view of patient recovery goals. These professionals play a fundamental role in the context of patient recovery and are involved in all dimensions of the process^(32)^.

In practice, it is pertinent to distinguish the roles of members of the multidisciplinary team, and the results showed that both theories and conceptual frameworks contributed to articulating the nursing role in the care environment. In this regard, nurses work in acute stroke services and perform assessment, identification, and monitoring actions, as well as rehabilitation for survivors^(33)^.

Another essential point on which the nursing team can act is the process of transition and adaptation in the post-stroke period, where the individual has to adapt to the hospital environment and routines. Thus, it was found that Roy’s adaptation theory and Meleis’ theory of transitions can support nursing care in this aspect^(20,23)^. Additionally, it is crucial that while the patient receives care in the hospital setting, they are prepared for self-management of their recovery after discharge. Successful transitions are emphasized as a fundamental competence of nursing^(34)^.

The results highlight the importance and validate the value that theories and conceptual frameworks have in implementing effective care plans to optimize outcomes for stroke survivors. Patients who received interventions built on nursing theories showed better outcomes related to cognitive function, neurological function, and activities of daily living^(17,19)^.

No practical theory supporting nursing care within the studied context was identified, which suggests that little knowledge has been produced to support practice based on a nursing theory. In this sense, it is understood that theory and practice should go hand in hand, but in the country, studies that value the potential of nursing theories are still in the minority^(8)^.

Finally, it is emphasized that the application and integration of nursing theories in various contexts are necessary for advancing the knowledge of nursing science. In the practical context, nurses who understand the conceptual meanings of their know-how legitimize and support their care, fostering the development of their profession^(35)^.

### Study limitations

This study had limitations concerning the number of databases searched, which may have restricted access to additional data. Another limiting factor was the specific research context, as numerous studies addressing the research objective were identified in a community setting rather than encompassing hospital care. Lastly, the selected strategy for evaluating the included records indicated that most of them were classified as having a low level of evidence.

### Contributions to the Field

The findings hold significance for nursing professionals as they enable an understanding of how theoretical constructs were employed to facilitate the appropriate development of care for stroke patients, bridging the gap between theoretical and practical realms and contributing to safe, efficient, and high-quality nursing care. Furthermore, this study may encourage the development of specific theories tailored to the researched context, as no theories were identified that were specifically designed for this purpose. Additionally, it provides additional information and strategies that can be implemented in clinical practice to enhance the patient’s experience during their recovery process.

## FINAL CONSIDERATIONS

This scoping review successfully mapped the evidence regarding nursing theories applied to the care of stroke patients. Major theories, mid-range theories, and conceptual frameworks were identified for this purpose. It is crucial to emphasize that this review specifically focused on care for stroke patients in the hospital setting, considering appropriate treatment assistance, evaluation of attention needs across various dimensions, as well as the adaptation and transition of care for the patient.

Thus, it was possible to ascertain that the theoretical constructs evidenced through theories and conceptual frameworks serve as facilitators and enhancers of nursing interventions. Moreover, they provide more precise and organized guidance for the care of stroke patients. However, the results also underscore the need for further research that highlights this field of nursing knowledge and supports professionals in their practice.
